# The Dynamics of Sex Ratio Evolution: From the Gene Perspective to Multilevel Selection

**DOI:** 10.1371/journal.pone.0060405

**Published:** 2013-04-17

**Authors:** Krzysztof Argasinski

**Affiliations:** Institute of Environmental Sciences, Jagiellonian University, Kraków, Poland; Vrije Universiteit, Netherlands

## Abstract

The new dynamical game theoretic model of sex ratio evolution emphasizes the role of males as passive carriers of sex ratio genes. This shows inconsistency between population genetic models of sex ratio evolution and classical strategic models. In this work a novel technique of change of coordinates will be applied to the new model. This will reveal new aspects of the modelled phenomenon which cannot be shown or proven in the original formulation. The underlying goal is to describe the dynamics of selection of particular genes in the entire population, instead of in the same sex subpopulation, as in the previous paper and earlier population genetics approaches. This allows for analytical derivation of the unbiased strategic model from the model with rigorous non-simplified genetics. In effect, an alternative system of replicator equations is derived. It contains two subsystems: the first describes changes in gene frequencies (this is an alternative unbiased formalization of the Fisher-Dusing argument), whereas the second describes changes in the sex ratios in subpopulations of carriers of genes for each strategy. An intriguing analytical result of this work is that the fitness of a gene depends on the current sex ratio in the subpopulation of its carriers, not on the encoded individual strategy. Thus, the argument of the gene fitness function is not constant but is determined by the trajectory of the sex ratio among carriers of that gene. This aspect of the modelled phenomenon cannot be revealed by the static analysis. Dynamics of the sex ratio among gene carriers is driven by a dynamic “*tug of war*” between female carriers expressing the encoded strategic trait value and random partners of male carriers expressing the average population strategy (a primary sex ratio). This mechanism can be called “*double-level selection*”. Therefore, gene interest perspective leads to multi-level selection.

## Introduction

Sex ratio evolution is one of the basic examples of evolutionary mechanisms that are presented in every course on evolutionary biology. The first approach to this problem was presented by German biologist Carl Dusing [2]. Historically, it was the first application of mathematical modeling to evolutionary phenomena. Dusing argued that the fitness of females using different sex ratio strategies can be described by the number of their grandoffspring. A similar approach was applied by Fisher and Shaw and Mohler [3], [4], [5]. This is also an important example in evolutionary game theory, known as a *sex ratio game* [6], [7], [8], [9], [10], [11]. The general prediction of this approach is that the sex ratio of 0.5 is evolutionarily stable. However, there is an alternative approach to the modeling of sex ratio evolution related to population genetics [5], [12], [13], [14]. This approach is focused on tracing the genes encoding sex ratio strategies. Those models predict a stable structure of the population describing gene frequencies among males and females and a sex ratio as the effect of expression of those genes. Therefore, there is a major difference between the strategic phenotypic approach and genetic modeling [15], [16], [17]. The phenotypic approach describes the mean female strategy of 0.5 as evolutionarily stable, while genetic models show that the composition of the male population can also matter. To analyze this problem, in our previous paper [1], a new model of sex ratio evolution was developed. The new approach is an attempt to combine the genetic and phenotypic approach and to overcome the limitations of both of them. The goal was to solve the problem of different predictions and to obtain a coherent picture of the modeled phenomenon.

The new model focuses on the global dynamics of the system, and its structure resembles the genetic approach [5], [12], [13], [14]. Whereas the classical Dusing-Fisher-Shaw-Mohler (DFSM) model is focused on the reproductive success of individual strategies carried by female strategic agents (as in Dusing's paper, see [2], or the sex ratio game) or some undescribed group of “parents” (as in [3], [4], more on this topic in section 4.2). For a closer understanding of the relations between the classical and the new approach, the selection of individual strategies resulting from global dynamics must be analyzed, which is the subject of this paper.

In this paper a novel technique of change of coordinates will be applied to the model from [1]. This will reveal new aspects of the modelled phenomenon which cannot be shown or proven in the original formulation. Similarly the results from [1] will be hard to show in the new coordinates, thus the two papers complement each other. The underlying goal is to describe the dynamics of selection of particular genes in the entire population, instead of in the same sex subpopulation as in the previous paper and earlier population genetics approaches. In effect, an unbiased strategic model will be analytically derived from the non-simplified rigorous genetic model.

Thus, the classical strategic approach analyzes the reproductive success of a female, while the genetic approach traces gene frequencies in the population. Therefore, what happens when we combine both perspectives and assume that the gene is the strategic agent?

## Methods

Now we shall recall the structure of the new model (see Table 1 for the list of symbols). Section 1 can be skipped by readers familiar with paper [1].

**Table 1:** List of important symbols:

classical theory:
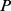 – secondary sex ratio
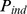 – individual strategy interpreted as the mean sex ratio in the brood of a single female, which is the carrier of this strategy ( 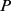 with index denotes the individual strategy)
*N*- population size
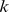 – mean brood size of a single female
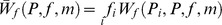 – mean fitness function of the female subpopulation
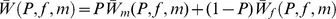 – mean fitness function of the whole population
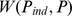 – classical Dusing-Fisher-Shaw-Mohler fitness function
**new model:**
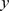 – number of males
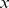 – number of females
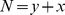 – population size
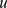 – number of individual strategies
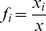 – frequency of females with strategy 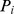
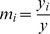 frequency of males with strategy 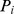
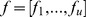 -state vector of the female subpopulation
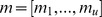 -state vector of the male subpopulation
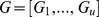 – state vector of the gene pool
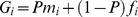 - frequency of a gene which encodes the strategy 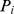
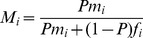 fraction of males in the subpopulation of carriers of the strategy 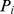
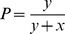 – frequency of males in the population
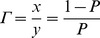 – number of females per single male individual
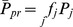 -primary sex ratio (mean strategy in the female subpopulation)
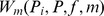 – males' payoff function
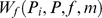 – females' payoff function
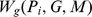 - fitness function of a gene which encodes strategy 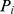
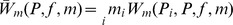 – mean fitness function of the male subpopulation

### 1.1 Summary of basic formal details of the new model

There are 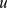 individual strategies described by 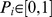, the proportion of male offspring of a female playing strategy 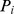. There are 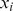 females and 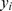 male carriers of the strategy 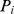 in the population. Therefore, the population consists of 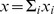 females and 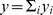 males. Thus, 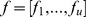 is the vector of frequencies of strategies of the female subpopulation, and 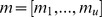 is an analogous vector for the male subpopulation, where 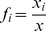 and 
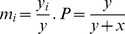
 is the fraction of males in the population (the secondary sex ratio), and 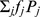 is the mean female strategy (the primary sex ratio). Assume that each female produces 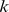 offspring according to haploid inheritance. However, males are gene carriers too, and transfer those genes to their offspring with the probability 0.5. The influence of males can be described by the *fitness exchange effect* (i.e. daughters of male carriers contribute to the fitness of female carriers and sons of female carriers contribute to the fitness of male carriers). In [1] it was shown that 
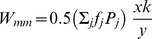
 is the expected number of male offspring, and 
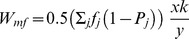
 is the expected number of female offspring of the male individual. Analogously, 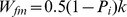 is the expected number of male offspring, and 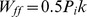 is the expected number of female offspring of the female individual playing the strategy 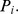 Therefore, the following equations were obtained:
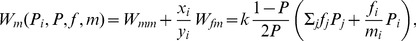
(1)– payoff function of the males carrying the strategy 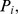


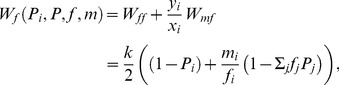
(2)– payoff function of the females playing the strategy 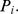


Now we have all elements needed to formulate multipopulation replicator dynamics (see appendix A in the File S1). In [1], this took the following form:


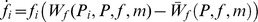
 for 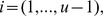



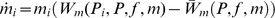
 for 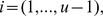


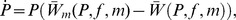
where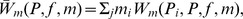 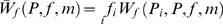 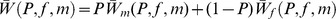 are the respective average payoff functions of the male, female and the whole population. This leads to the following system of equations:


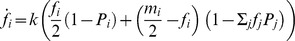
 for 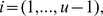



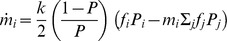
 for 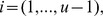


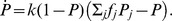



It was shown that, for biological reasons, we can limit the analysis of the model to values of primary and secondary sex ratios over the interval 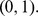


### 1.2 Summary of predictions of the new model

An analysis of the behavior of this model shows that two phases of convergence can be distinguished. The first, rapid phase occurs when the secondary sex ratio 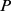 converges to the current value of the primary sex ratio 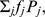 and the male subpopulation converges to the state termed the male subpopulation equilibrium (MSE), described by the condition 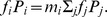 During the second phase of convergence, the primary sex ratio converges to the value 0.5, and the value of the secondary sex ratio follows these changes to maintain equality. In addition, the state of the male subpopulation changes to maintain the MSE.

## Results

### 2. Reformulation of the model

In the previous paper [1], a change in the coordinates (described in appendix A in the File S1) was applied to the numerical solutions obtained to calculate the frequencies of all types of individuals (see Fig. 3c in [1] and section 3.2 there) and gene frequencies (see Fig. 6 in [1] and section 4 there). However, this method can be applied not only to numerical solutions, but also directly to replicator equations. In this way, we can reformulate the new model to focus on changes in gene frequencies. We have 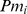 male carriers and 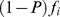 female carriers of a strategy 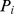 in the whole population. Thus, the frequency of carriers of a gene which encodes this strategy is equal to:
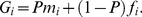
(3)


**Figure 3:**
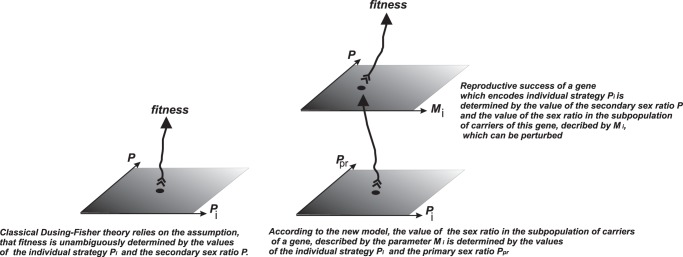
A comparison of “single level” selection and “double level” selection.

The state of the population can be described by the vector 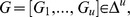 where 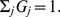 In this description, there is no information about the sex of the carriers of these genes. We can fill this gap by adding information about the sex ratio in the subpopulation of the carriers for every gene:


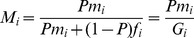
 -proportion of males among carriers of 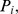




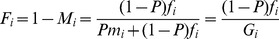
(4)-proportion of females among carriers of 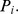


**Figure 1:**
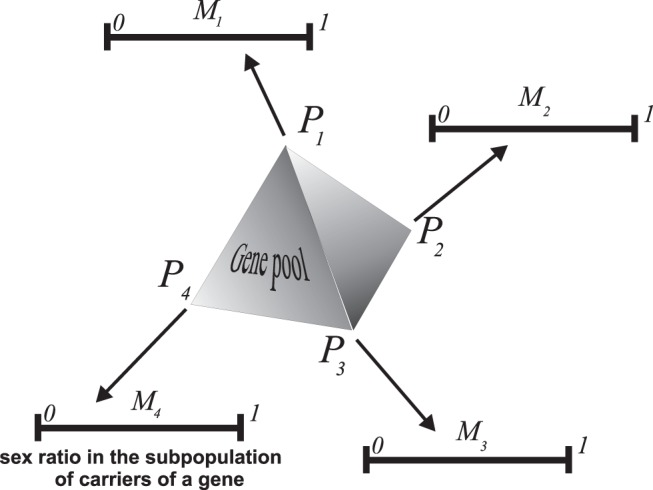
Scheme of a space of population states in the new formulation of the model. In this case, it is a product of a simplex of gene frequencies and 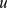 one-dimensional simplexes that describe sex ratios in the subpopulations of carriers for each strategy.


Then, 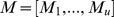 is the vector of subpopulation sex ratios. Therefore, this structure can be treated as a division of the entire population into 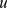 subgroups with one-dimensional subpopulation states. Then, according to the general notation from appendix A in the File S1, 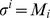 and 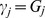 (see also [18]), the structure of the space of population states will take the form presented in Fig. 1. Note that in the previous formulation of the model, the space of population states was the product of two 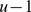 dimensional simplexes of the male and female subpopulation and a one-dimensional simplex of the proportion between these subpopulations (a secondary sex ratio); in general, the dimension of the whole space was 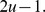 In the new formulation, this space consists of one 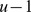 dimensional simplex of gene frequencies and 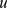 one-dimensional simplexes of subpopulation sex ratios, and the dimension of the whole space of population states is also 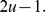 Therefore, the dimension of the space of population states is invariant in response to the change of coordinates, which is consistent with the fact that we have a different parameterization of the same phase space. We can describe important population parameters in the new coordinates for parameters such as the mean female subpopulation strategy 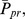 i.e., the primary sex ratio and secondary sex ratio (among adult individuals) 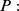


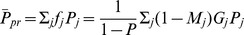
 and 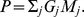


The average fitness functions from the previous paper (recalled in section 1.1) were:


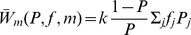
 – mean fitness of the male subpopulation,


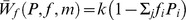
 – mean fitness of the female subpopulation,


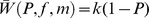
 – mean fitness of the whole population.

Then, we can derive the mean payoff to the carrier of a gene for strategy 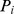 (for a full derivation see appendix B in the File S1):
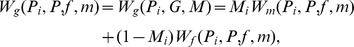
which takes the form:
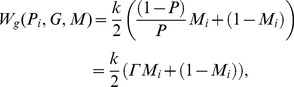
(5)where 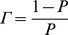 is the number of females per single male individual. For the new coordinates we obtain the following replicator equations (for a detailed derivation, see appendix C in the File S1):


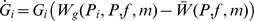
 -dynamics of gene frequencies,


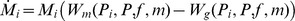
 -dynamics of sex ratios in carriers subpopulations,which take the form:



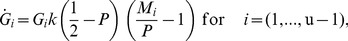
(6)




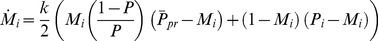
(7)

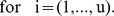


### 3. Behavior of trajectories of replicator equations

#### 3.1 Trajectories of gene frequencies

Here, we will examine the dynamics of gene frequencies. The product 
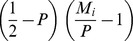
 is responsible for the sign of the right side of equation (6). When both coefficients are negative or positive, then their product is positive (the frequency of gene 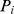 increases), and when they have opposite signs, then their product will be negative (the frequency of gene 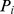 decreases). The zero points of these coefficients, 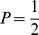 and 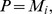 are stationary points of equation (6). Therefore, the dynamics of the gene frequencies can be described in the following way:


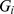
 increases when 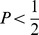 and 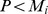 or 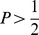 and 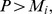



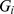
 decreases when 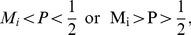
(8)


 



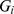
 is constant when 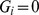 or 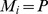 or 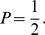


**Figure 2:**
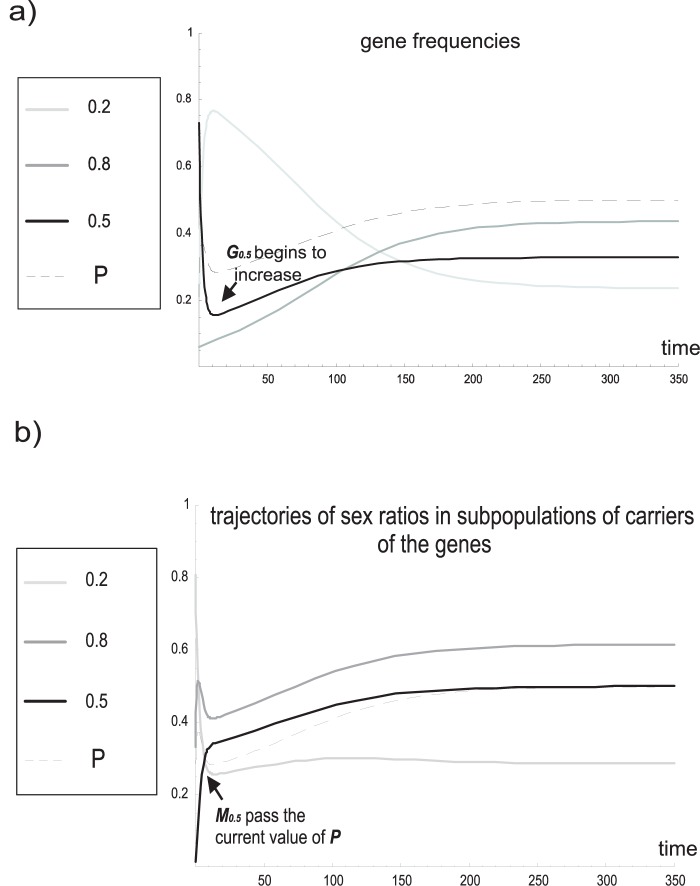
Trajectories of a population of individuals with strategies for sex ratios of *0.2, 0.5* and *0.8* for initial conditions. 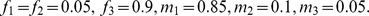
 Panel *a*) shows the trajectories of gene frequencies 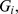 Therefore, 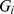 increases when 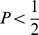 and 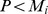 or 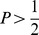 and 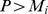 and decreases when 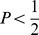 and 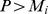 or 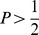 and 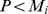. This mechanism is clearly shown in the trajectories of strategy *0.5*. The trajectory 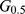 switches from a decrease to an increase when trajectory of 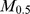 passes the trajectory of 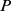 (see panel *b*). Panel *b*) shows the respective changes of sex ratios in carrier subpopulations 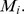 Note that sex ratios in carrier subpopulations rapidly converge to the values determined by the MSE phenomenon, and after that, they follow the changes of the primary sex ratio 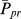 that slowly converges to *0.5*. The sex ratio among carriers of male biased strategies change due to the dynamics of the primary sex ratio while among female biased strategies, it converges to the neighbourhood of the value encoded by the gene.

Recall that 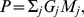 which means that the secondary sex ratio is equal to the average sex ratio in the carrier subpopulation over the entire population. Therefore, the frequency 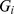 decreases when the sex ratio in the carrier subpopulation 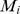 is shifted farther from 0.5 than the mean sex ratio in the carrier subpopulations for all strategies 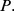 In the opposite case, 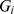 will increase. This mechanism is illustrated in Fig. 2a. Therefore, the frequency of a gene that encodes the strategy 0.5 increases when the sex ratio in a subpopulation of its carriers is closer to 0.5 than the current value of the secondary sex ratio; this frequency decreases in the opposite case. A situation in which the secondary sex ratio is equal to 0.5 is the stationary state of the dynamics of gene frequencies (6). Therefore, this mechanism described by (8) is independent of individual strategies *P_i_*, but its dynamics are dependent on the trajectories of the sex ratios in the subpopulations of carriers of the strategies described by *M_i_*. Note that parameter *M_i_* also affects the secondary sex ratio 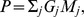 modifying the values of 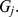 However, sex ratios in carrier subpopulations 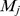 are determined by mechanisms acting at the level of carrier subpopulations that are described in the next section.

#### 3.2 Trajectories of sex ratios in subpopulations of carriers

The dynamics of sex ratios in the carrier subpopulations are more sophisticated. The right side of equation (7) contains two coefficients: 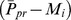 and 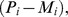 weighted by current values of 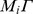 and 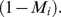 These coefficients are responsible for the direction of convergence. The coefficient 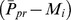 induces attraction of 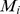 to 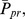 and the coefficient 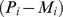 causes attraction of 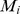 to 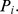 This is, in a sense, a *tug of war* between female partners of the male carriers (representing average strategy 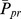) and female carriers of the same gene (representing encoded strategy 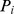). As we can see in Fig. 2b, the shape of the trajectory of a 0.8 sex ratio strategy that produces mostly sons is almost parallel to the trajectory of parameter 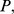 which is equal to 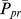 in the slow phase of convergence (see [1]). On the other hand, the trajectory of a 0.2 sex ratio strategy that produces more daughters is closer to the constant function 0.2 than to the trajectory of 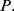 Thus, the *M_i_* value of the strategies producing (and in effect carried by) mostly males resemble trajectories of the primary sex ratio, while female biased strategies have *M_i_* almost constant and equal to *P_i_*. This interesting aspect would be hard to show by static analysis. Below, we will characterize equilibrium in this “tug of war”.

Lemma 1

- For every set of values of 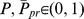 and 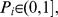 dynamics (7) has the unique stable conditional equilibrium 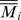 that is contained in the interval limited by the values of 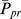 and 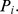

- For the strategy 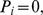 there is one stationary point, 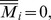 which is stable when unique. However, when 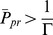 and 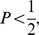 the rest point 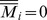 becomes unstable, and there exists a second stationary point 
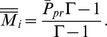

For a proof, see Appendix D in the File S1.

Lemma 1 indicates that, at every moment, there exists some attracting point for *M_i_* lying between the current value of the primary sex ratio 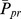 (which also changes in time) and the value of individual strategy *P_i_*. By this dynamic equilibrium, the expression of individual strategies determines the parameter *M_i_*. The only exception is strategy 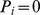 (production of female offspring only) for which the second stationary state may exist during the rapid phase of convergence. It was impossible to analytically derive the stable sex ratio in the carrier subpopulations, in the general case. This is possible only when the population is in the MSE state and will be presented in a subsequent paper devoted to the MSE. According to Lemma 1, we can numerically approximate this value because it is unique in these biologically significant cases.

## Discussion

### 4.1 The mechanism of “double-level” selection

Here, we will summarize the results we have obtained. The first intriguing analytical result of the reformulated model is that the fitness function of a gene (5) is independent of the individual strategy it encodes. Proliferation of a given gene depends on the current sex ratio in the subpopulation of its carriers 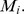 Note that the fitness function (5) is a good mathematical description of Fisher's idea, which is related to the reproductive value of carriers with different sexes according to the deviation of the secondary sex ratio *P*. It suggests that males are reproductively more efficient when they are in the minority (*P<1/2*), because each male can mate with several females (Γ>1). On the other hand, females are more efficient when they are in the minority (*P>1/2*), because each female will be expected to produce offspring, and there are not enough mates for all males (Γ<1). Therefore, parameter 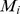 describes the proportion of carriers with the more reproductively efficient sex among all carriers of a gene. This fitness function explicitly considers male carriers from the mother's generation of unexpressed sex ratio genes. Function (5) can be transformed in the following way (recall that 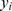 is the number of male carriers, and 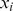 is the number of female carriers, of the strategy 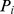):
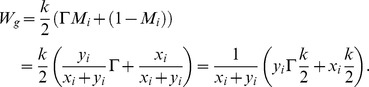



This is the per capita normalized sum (averaged over the carriers subpopulation) of the offspring produced by female partners of male carriers described by 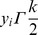 and offspring of female carriers described by 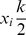 (where 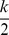 is the number of offspring of a single female multiplied by the probability of gene transfer from the focal parent). This is an explanation of the importance of male carriers of the unexpressed sex ratio genes, or rather their female partners. Their role is important, because each male carrier may have 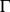 partners, and the activity of their partners is an important component of gene fitness. Surprisingly, this function is independent of the value of a given strategy, 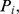 encoded by the carried gene. It depends only on 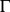 and 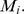 The phenomenon can be termed *double level selection*. The fitness of a gene that encodes an individual strategy is determined in some way by the current sex ratio in its carrier subpopulation and the secondary sex ratio in the population as a whole. Values of both parameters may be perturbed. However, the stable carrier subpopulation sex ratio should be determined in some way by the value of the encoded strategy (Fig. 3). This is a newly discovered mechanism. In general, the mechanism of double level selection can be regarded as an example of *multi-level selection*, which is the concept presented by [19], [20], [21], [22], [23]. The classical approach to the modeling of sex ratio evolution treats this phenomenon as *single level* selection, which means that the fitness is unambiguously determined by the values of individual strategy 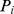 and a population state described by the secondary sex ratio (Fig. 3). In the next subsection, a higher level of this process will be considered.

### 4.2 Dynamics of gene frequencies

The mechanism realized by gene frequency replicator equations (6), described by the rules (8) increases the frequency of a gene for which the value of a parameter 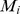 is greater/smaller than the secondary sex ratio *P* (which is equal to the average *M* in the population) when *P* is smaller/greater than 0.5. Thus, it is profitable for the gene to be carried by that sex which is currently in the minority. There is an interesting relationship between the mechanism described by (8) and the replicator dynamics paradigm. In standard replicator equations, frequencies of strategies change according to the sign of the deviation of their fitness from average fitness (minus – decrease, plus – increase). If fitness depends linearly on a particular trait, then selection works according to deviations from the average trait value. Note that the payoff function (5) is linear with respect to the parameter (trait) *M_i_*, and the secondary sex ratio *P* is an average *M_i_* over the population. The difference between the mechanism in rules (8) and standard replicator dynamics is that parameter 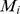 is not a description of a fixed individual strategy but of the current state of a subgroup of individuals (the subpopulation of carriers of strategy 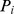). Dusing classically argued that female producing offspring of the sex that is currently in the minority will have more grand-offspring. This argument states that there are differences in fitness among females with different strategies, which is considered a proof of the existence of selection on individual strategies. However, our new model shows that a mechanism based on different reproductive values is independent of individual strategies 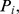 and it affects the primary sex ratio 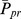 and the secondary sex ratio 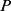 (which is equal to the average sex ratio in the carrier subpopulation) by changing only gene frequencies 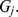 In [3], the following statement can be found:

*“...it would follow that those parents, the innate tendencies of which caused them to produce males in excess, would for the same expenditure, produce a greater amount of reproductive value; and in consequence would be the progenitors of a larger fraction of future generations…”.*

Therefore, Fisher in his original reasoning considered a group of individuals that adjusts the sex ratio among its members due to genetic mechanisms. However, the mechanisms for this adjustment were not explicitly explained. The perspective of a group adjusting the sex ratio among its members is also assumed by [4]. However, they also presented only a conjecture that the sex ratio is completely heritable within the group, without an explanation of how it is realized. Therefore, there is a difference between Fisher's reasoning that operated on the level of the subpopulation of all carriers of a gene and Dusing's approach related to the level of female individuals. The female perspective is not sufficient, especially for male-biased strategies, which will produce more male than female carriers. This means that the Fisherian argument about the different reproductive values of males and females is an important part of understanding sex ratio self-regulation. However, it is not enough for a full mechanistic explanation of this process. Therefore, we should investigate how the expression of individual strategies determines the sex ratio in the carrier subpopulation 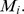 This will allow us to overcome the limitations of Dusing's reasoning, which considers only female reproductive success and disregards the role of male gene carriers from the same generation.

### 4.3 Dynamics of sex ratios in carrier subpopulations: the “tug of war” mechanism

The sex ratio in carrier subpopulations is the effect of intrinsic dynamics that can be compared to a “tug of war” between 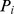 and 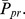 It was proved in Lemma 1 that for every population state there exists a single unique attractor of *M_i_* dynamics contained in the interval that is limited by values of 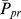 and 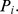 Let us describe the “tug of war” metaphor in a more formal way. The right-hand side of replicator equation (7) is proportional to
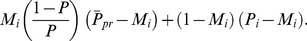



The factor 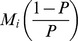 that is the weight of 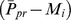 can be written as 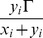 and the proportion 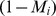 that is the weight of 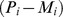 equals 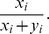 Thus the right side of this equation is proportional to
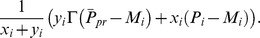



Since 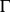 is the number of females per single male, then 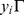 is also the number of female partners of male carriers of gene encoding the strategy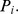 These females “pull the rope” toward the value of 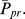 On the other side, a team of 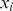 female carriers of this gene “pulls the rope” toward the value 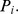 It is evident here that the expression of strategies of parental individuals determines the fate of their descendants, by the setting of the sex ratio among them.

### 4.4 An unresolved problem: the role of the male subpopulation equilibrium

Recall that, during the slow phase of the sex ratio dynamics, 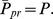 Note that, if in rules (8) we substitute 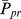 instead of 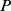 and 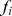 instead of 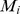 we obtain the following rules:


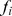
 increases when 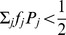 and 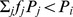 or 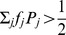 and 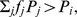



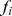
 decreases when 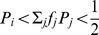 or 
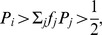



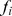
 is stable when: 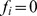 or 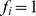 or 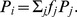


These describe the changes of a female subpopulation state when the MSE condition is satisfied (Lemma 1 from [1]). This leads to the problem of the role of the MSE phenomenon, which is responsible for the rapid phase of convergence and the dynamics of sex ratios in the carrier subpopulations. The first idea that comes to mind to explain this phenomenon is that the male subpopulation equilibrium is equivalent to some stable sex ratio in the carrier subpopulation (the equilibrium of the “tug of war” mechanism), which is conditional on current values of 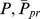 and 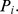 The rapid phase will then be equivalent to convergence to this stable value. When the subpopulation reaches a stable sex ratio, then it simply follows changes of the primary (and in effect the secondary) sex ratio, which are equivalent to the slow phase of convergence. Unfortunately, this idea is false. As shown in [1], when the MSE conditions are satisfied for all strategies, then all males in the population have the same fitness. If we assume that carrier subpopulations are in their stable states, then for all strategies females will have fitness equal to males. So, when all males have equal fitness, and all females have fitness equal to males, then all individuals in the population have equal fitness. In this case, the population would be in a global stationary state, which is not true. The nature and role of the male subpopulation equilibrium are the subjects of a subsequent paper.

## Supporting Information

File S1 **Appendices A–D.**
(DOC)

## References

[1] Argasinski K (2012) The dynamics of sex ratio evolution Dynamics of global population parameters. J Theor Biol 309: 134–146. 22683379 10.1016/j.jtbi.2012.05.025

[2] Edwards AWF (2000) Carl Dusing (1884) on the Regulation of the Sex-Ratio. Theor Pop Biol 58: 255–257. 11120652 10.1006/tpbi.2000.1482

[3] Fisher RA (1930) The Genetical Theory of Natural Selection. Oxford University Press (New Edition 2000). 298 p.

[4] Crow JF, Kimura M (1970) An introduction to Population Genetics Theory. Harper & Row. 608 p.

[5] Seger J, Stubblefield JW (2002) Models of Sex Ratio Evolution. In: Hardy ICW, editor. Sex Ratios, Concepts and Research Methods. Cambridge University Press. pp. 2–25.

[6] Bomze IM, Potscher BM (1989) Game theoretical foundations of evolutionary stability. Lecture Notes in Economic and Mathematical Systems. vol. 324, Springer. 151 p.

[7] Cressman R (1992) The Stability Concept of Evolutionary Game Theory. Springer. 128 p.

[8] Hofbauer J, Sigmund K (1988) The Theory of Evolution and Dynamical Systems. Cambridge University.Press. 352 p.

[9] Hofbauer J, Sigmund K (1990) Evolutionary Games and Population Dynamics. Cambridge University Press. 351 p.

[10] Weibull J (1995) Evolutionary Game Theory. MIT press. 265 p.

[11] Maynard Smith J (1982) Evolution and the theory of games. Cambridge University Press. 234 p.

[12] Karlin S, Lessard S (1986) Theoretical Studies on Sex Ratio evolution. Princeton University Press. 332 p. 3526135

[13] Eshel I, Feldman M (1982) On Evolutionary Genetic Stability of the Sex Ratio. Theor Pop Biol 21: 430–439.

[14] Eshel I, Feldman M (1982) On the Evolution of Sex Determination and the Sex Ratio in Haplodiploid Populations. Theor Pop Biol 21: 440–450.

[15] Oster GF, Rocklin SM (1979) Optimization models in evolutionary biology In: Levin SA, editor. Lectures on Mathematics in the Life Sciences. vol. XI. AMS. 21–88.

[16] Bomze IM, Shuster P, Sigmund K (1983) The role of Mendelian genetics in strategic models on animal behavior. J Theor Biol 101: 19–38. 6876824 10.1016/0022-5193(83)90271-0

[17] Weissing FJ (1996) Genetic versus phenotypic models of selection: can genetics be neglected in a long-term perspective? J Math Biol 34(5): 533–555. 8691084 10.1007/BF02409749

[18] Argasinski K (2006) Dynamic multipopulation and density dependent evolutionary games related to replicator dynamics. A metasimplex concept. Math Biosci 202: 88–114. 16797041 10.1016/j.mbs.2006.04.007

[19] Wilson DS, Van Vugt M, O'Gorman R (2008) Multilevel selection theory and major evolutionary transitions: implications for psychological science. Current Directions in Psychological Science. 17 (1): 6–9.

[20] Wilson DS (1997) Introduction: Multilevel selection theory comes of age. The Am Nat 150(S1): 1–21. 18811307 10.1086/286046

[21] Wade MJ, Wilson DS, Goodnight C, Taylor D, Bar-Yam Y, et al.. (2010) Multilevel and kin selection in a connected world. Nature. 463(7283) E8–E9. 10.1038/nature08809 PMC3151728 20164866

[22] Traulsen A, Nowak MA (2006) Evolution of cooperation by multilevel selection. PNAS 103(29): 10952–10955. 16829575 10.1073/pnas.0602530103 PMC1544155

[23] Damuth J, Heisler IL (1988) Alternative formulations of multilevel selection. Biology and Philosophy 3(4), 407–430.

