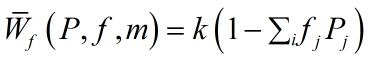# Correction: The Dynamics of Sex Ratio Evolution: From the Gene Perspective to Multilevel Selection

**DOI:** 10.1371/annotation/38d9479a-d3be-4ef4-8a7f-3431c7ae1db0

**Published:** 2013-12-13

**Authors:** Krzysztof Argasinski

There are incorrectly formatted formulas in Table 1. Please see the corrected Table 1 here: 

**Figure:**
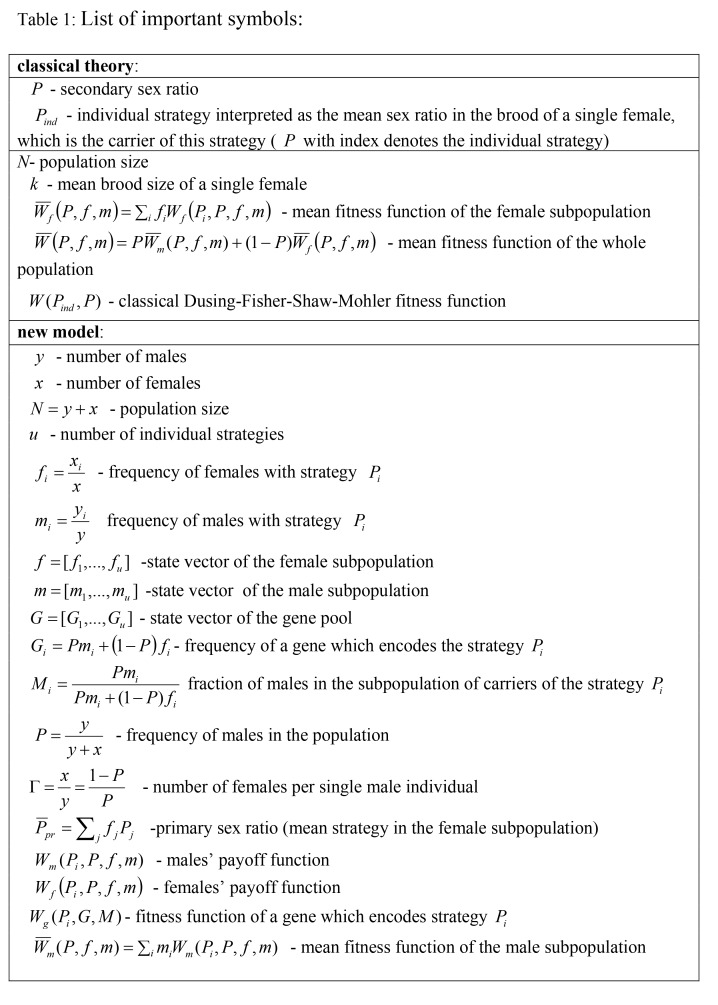


The equations immediately after Equation 2 should appear on separate lines. Please see the corrected equations here: 

**Figure:**
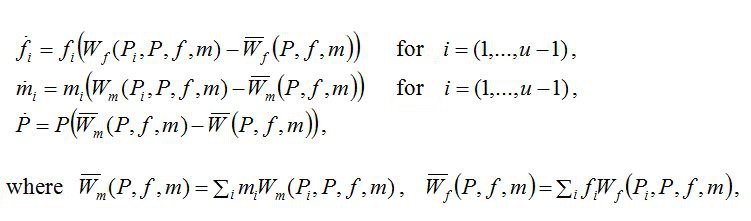


The equation for the 'mean fitness of the female subpopulation' under the Results section is incorrect. Please see the corrected equation here

**Figure:**