# Supplementary material for: The Dynamics of Sex Ratio Evolution: From the Gene Perspective to Multilevel Selection
Source: PLoS One. 2013 Apr 17;8(4):e60405. doi: 10.1371/journal.pone.0060405 (PMC3629214; doi:10.1371/journal.pone.0060405)
Supplement: File S1 — Appendices A–D. (DOC) [file pone.0060405.s001.doc]

|  |
| --- |

Appendix A

Change of coordinates in the space of population states.

Assume that we want to break down an entire population into $z$ subgroups. Define $d^{i}=[d_{1}^{i},..., d_{u_{i}}^{i}]$ as a vector of indices of strategies exhibited by individuals from the $i$-th subgroup ( $d_{j}^{i}\in\{1,..., u\}$, $u_{i}$ the number of strategies in the $i$-th subgroup). For example, the notation $d^{2}=[1, 3, 5]$ means that in the second subgroup, there are individuals with strategies $1, 3$ and $5$. Every strategy should belong to a single unique subgroup (and cannot belong to two). Then, according to [18] using the following change of coordinates:

$\sigma^{i}=[\sigma_{1}^{i},..., \sigma_{u_{i}}^{i}]=\left[\frac{\sigma_{d_{1}^{i}}}{\sum_{j=1}^{u_{i}} \sigma_{d_{j}^{i}}},..., \frac{\sigma_{d_{u_{i}}^{i}}}{\sum_{j=1}^{u_{i}} \sigma_{d_{j}^{i}}}\right]$ for $i=1,..., z$, (a1)

we obtain the distribution of relative frequencies of strategies in the $i$-th subpopulation. The distribution of proportions between subpopulations has the form:

$\gamma=[\gamma_{1},..., \gamma_{z}]=\left[\sum_{i=1}^{u_{1}} \sigma_{d_{i}^{1}},..., \sum_{i=1}^{u_{z}} \sigma_{d_{i}^{z}}\right]$, (a2)

where $\gamma_{i}$ is the proportion of the $i$-th subpopulation. Every decomposition into subpopulations can be reduced again to a single population model by the opposite change of coordinates $\sigma(\gamma, \sigma^{1},..., \sigma^{z})$ where:

$\sigma_{d_{j}^{i}}=\gamma_{i}\sigma_{j}^{i}$. (a3)

Note that we can break down an entire population into $z$ subpopulations. When we apply the above transformations to replicator equations, we obtain a set of equations that describes the dynamics inside subpopulations (intraspecific dynamics, see [18]), which has the form:

, where $W_{j}$ is the fitness of the $j$- th strategy in the $i$- th subpopulation and $\bar{W}^{i}$ is the mean fitness in the $i$- th subpopulation, and a system that describes changes of relative sizes among subpopulations (interspecific dynamics) is:

, where $\bar{W}$ is the mean fitness in the whole population.

When the set of strategies in each subpopulation is characterized by a vector of indices $d^{i}$, then the system of replicator equations will be:

for $j=1,..., u_{i}-1$ and $i=1,..., z$, (a4)

for $s=1,..., z-1$, (a5)

where $\bar{W}^{s}(\sigma)=\sum_{i=1}^{u_{s}} \sigma_{i}^{s}W(P_{d_{i}^{s}},\sigma( \gamma, \sigma^{1},..., \sigma^{z}))$ is the mean fitness in the $s$-th subpopulation. The argument of a fitness function is a set of relative frequencies of all individuals $\sigma$ (without division into subpopulations), therefore the opposite change of coordinates $\sigma(\gamma, \sigma^{1},..., \sigma^{z})$ (a3) should be applied ([18]). In practical applications of this method to the modeling of biological problems, replicator equations can be defined for broken down populations. This break down will simplify the formulation of the model because, when strategies are initially assigned to subpopulations, there is no need to change their indices. The choice of subpopulations is arbitrary and depends on the biological assumptions underlying the analyzed problem. The entire population may be divided into two competing subpopulations of carriers and parasites or predators and prey. It may also be divided into two subpopulations of males and females, in which case interspecific dynamics will describe the evolution of the secondary sex ratio, and intraspecific dynamics will describe changes of frequencies of strategies inside male and female subpopulations. The entire population can be divided into more than two subpopulations. The subpopulations can be divided into sub-subpopulations, and the entire population may be transformed into a complex multilevel cluster structure. However, all of these structures are equivalent to a single population replicator dynamics model.

Appendix B

Derivation of the fitness function of a gene

$$W_{g}(P_{i}, P,f, m)=W_{g}(P_{i}, G, M)=M_{i}W_{m}(P_{i}, P,f, m)+(1-M_{i})W_{f}(P_{i}, P,f, m)=$$

$$=\frac{Pm_{i}}{G_{i}}W_{m}(P_{i}, P,f, m)+\frac{(1-P)f_{i}}{G_{i}}W_{f}(P_{i}, P,f, m)=$$

$$=\frac{k(1-P)}{2G_{i}}\left(m_{i}\left(\sum_{j} f_{j}P_{j}+\frac{f_{j}}{m_{j}}P_{i}\right)+f_{i}\left(\left(1-P_{i}\right)+\frac{m_{i}}{f_{i}}\left(1-\sum_{j} f_{j}P_{j}\right)\right)\right)=$$

$$=\frac{k(1-P)}{2G_{i}}\left(m_{i}\sum_{j} f_{j}P_{j}+f_{i}P_{i}+f_{i}\left(1-P_{i}\right)+m_{i}\left(1-\sum_{j} f_{j}P_{j}\right)\right)=$$

$$=\frac{k(1-P)}{2G_{i}}\left(m_{i}\left(\sum_{j} f_{j}P_{j}+1-\sum_{j} f_{j}P_{j}\right)+f_{j}\left(P_{i}+1-P_{i}\right)\right)=$$

$$=\frac{k(1-P)}{2G_{i}}\left(m_{i}+f_{j}\right)$$

The obtained formula should be described in new coordinates. Since:

$m_{i}=\frac{M_{i}G_{i}}{P}$ and $f_{i}=\frac{(1-M_{i})G_{i}}{1-P}$,

in effect we obtain:

$$W_{g}(P_{i}, G, M)=\frac{k(1-P)}{2G_{i}}\left(\frac{M_{i}G_{i}}{P}+\frac{(1-M_{i})G_{i}}{1-P}\right)=$$

= $\frac{k}{2}\left(M_{i}\frac{(1-P)}{P}+(1-M_{i})\right)$.

Appendix C

Alternative formulation of the replicator dynamics

Derivation of replicator equations:

a) Dynamics of gene frequencies (6):

$$=G_{i}k\left(\frac{\left(1-M_{i}\right)}{2}+\left(1-P\right) \left(\frac{M_{i}}{2P}-1\right)\right)=G_{i}k\left(\frac{1}{2}-\frac{M_{i}}{2}+\frac{1-P}{P}\frac{M_{i}}{2}-1+P\right)=$$

$$=G_{i}k\left(\left(\frac{1}{P}-2\right) \frac{M_{i}}{2}-\frac{1}{2}+P\right)=G_{i}k\left(\frac{1-2P}{P}\frac{M_{i}}{2}-\frac{1-2P}{2}\right)=G_{i}k\frac{1-2P}{2}\left(\frac{M_{i}}{P}-1\right)=$$

$=G_{i}k\left(\frac{1}{2}-P\right) \left(\frac{M_{i}}{P}-1\right)$.

b) Dynamics of sex ratios in carriers subpopulations (7):

.

Since $\frac{f_{i}}{m_{i}}=\frac{(1-M_{i})P}{M_{i}\left(1-P\right)}$ we have:

.

Then equation has the form:

.

In effect, we obtain an alternative set of replicator equations (6) and (7).

Appendix D

Proof of Lemma 1

The equation of the sex ratio in the carrier subpopulations (7) can be denoted:

. (d1)

At the stationary point, the right side of the equation should be equal to zero. The right side of this equation is a square polynomial of parameter $M_{i}$, then there exists at most two stationary points. Two terms are responsible for changing the direction of convergence: and $\left(P_{i}-M_{i}\right)$ weighted by the current values of $M_{i}$ and $\left(1-M_{i}\right)$. They are responsible for the attraction of $M_{i}$ suitably toward $\bar{P}_{pr}$ and $P_{i}$. If the current value of $M_{i}$ is smaller or larger than both values of $\bar{P}_{pr}$ and $P_{i}$, then both coefficients will have the same sign. If $\bar{P}_{pr}\neq P_{i}$, then both coefficients cannot attain zero in the same point. $M_{i}\in[0.1]$, and so it is obvious that the point that will zero the right side of equation should be contained in the interval limited by values of $\bar{P}_{pr}$ and $P_{i}$, because the terms will have opposite signs. It is also obvious that two stationary points cannot exist in the interior of the interval [0,1], because one should be an attractor and the second a repeller. This implies the existence of a third stationary point, which will be an attractor in the interval limited by a repeller and a boundary of the set [0,1]. Otherwise, the trajectory will escape the unit interval.

The interior has been analyzed. Thus we have to check the boundary of a set [0,1] where, the second stationary point, a repeller, may exist. This may be 0, when $\bar{P}_{pr}$ or $P_{i}$ is equal to 0, or 1, when $\bar{P}_{pr}$ or $P_{i}$ is equal 1. Values of $\bar{P}_{pr}$ from a boundary of [0,1] are not biologically relevant [1], therefore we have to review two cases:

a)$P_{i}=1$ and the possible restpoint $M_{i}=1$.

b) $P_{i}=0$ and the possible restpoint $M_{i}=0$.

When we substitute $M_{i}=1$ into a replicator equation , then $P_{i}$ vanishes, and the right side of equation (7) has a negative value, so this point is not stationary.

Thus, point a) is proven.

In the second case, when we substitute $M_{i}=0$ to the equation (d1), we obtain:

,

which means that for $P_{i}=0$, there exists a stationary point in the boundary. Then, in general, for $P_{i}=0$ equation (d1) takes the form:

. (d2)

Therefore, there are two cases, $M_{i}=0$ and , for which the right side of the equation can go to zero. The second stationary point is . Bracketed term in (d2) is negative with respect to M_i only for $\Gamma>1$, thus only in this case is stable. So we must check the following condition:

. (d3)

Thus for $\Gamma>1$when $\bar{P}_{pr}\leq1$(relevant case) and for $\Gamma<1$when $\bar{P}_{pr}\geq1$ (irrelevant case). Thus, for the case $\Gamma>1$, condition should be checked. This leads to the condition $\bar{P}_{pr}>\frac{1}{\Gamma}$.

After substitution of $\Gamma=\frac{1-P}{P}$ into obtained conditions we obtain:

$\bar{P}_{pr}>\frac{P}{1-P}$ and

When we parameterize $P=\frac{1}{a}$ where $a\in(1,\infty)$, we obtain:

$\bar{P}_{pr}>\frac{\frac{1}{a}}{1-\frac{1}{a}}=\frac{1}{a-1}>\frac{1}{a}$ (which means $\bar{P}_{pr}>P$)

and

.

So this phenomenon is structurally stable, however, it exists only when $P<\frac{1}{2}$ and parameter $P$ is shifted from the current value of $\bar{P}_{pr}$. This means that it may be observed only at the beginning of convergence to the male subpopulation equilibrium (a rapid phase). Which is the proof of point b).
